# Supplementary material for: Diversity patterns of the South African azooxanthellate scleractinians (Cnidaria: Anthozoa), with considerations of environmental correlates
Source: PLoS One. 2024 Aug 8;19(8):e0296188. doi: 10.1371/journal.pone.0296188 (PMC11309500; doi:10.1371/journal.pone.0296188)
Supplement: Attachment — Submitted filename: Peirano rev. PONE-D-23-17495_reviewer.pdf [file pone.0296188.s002.pdf]

# PLOS ONE

## The application of historic sample-specific variables in evaluating the biodiversity patterns of the South African azooxanthellate scleractinians (Cnidaria: Anthozoa).

--Manuscript Draft--

| | |
|---|---|
| **Manuscript Number:** | PONE-D-23-17495 |
| **Article Type:** | Research Article |
| **Full Title:** | The application of historic sample-specific variables in evaluating the biodiversity patterns of the South African azooxanthellate scleractinians (Cnidaria: Anthozoa). |
| **Short Title:** | Evaluating biodiversity patterns of South African azooxanthellate scleractinians. |
| **Corresponding Author:** | ZOLEKA PATRICIA NONTLANTLA FILANDER, MSc<br>Department of Environmental Affairs and Tourism: Republic of South Africa<br>Department of Forestry Fisheries and the Environment<br>Cape Town, Western Cape SOUTH AFRICA |
| **Keywords:** | stony Cold-water corals, longitude, depth, gradients, distribution. |
| **Abstract:** | Azooxanthellate scleractinian corals, a group of species that lack a symbiotic relationship with dinoflagellates, are influenced by environmental variables at various scales. As the global commitment to sustainably manage ocean ecosystems and resources rises, there is a growing need to describe biodiversity trends in previously unsampled areas. Benthic invertebrate research in South Africa is a developing field, and many taxa in deep water environments remain inadequately characterized. Recently, the South African azooxanthellate scleractinian fauna was taxonomically reviewed, but their distributional correlation with physical parameters have not been studied. Here we aim to understand the biodiversity gradients of the South African azooxanthellate coral fauna by analysing the environmental correlates of museum samples. The associated coordinate data were georeferenced and depth obtained from a national bathymetric dataset, prior to undertaking a multivariate analysis. Overall, our results confirmed two longitudinal groups (eastern margin [group A] vs southern and western margin [group B]) and 11 depths represented within two bathymetric zones (shallow [50-200 m] and deep [300-1000 m]). Both the longitudinal groups and depth zones partially explained coral distribution patterns, with depth highly correlated with species variation. Caryophylliids, flabellids, and dendrophylliids contributed the most towards distinguishing longitudinal and depth gradients. Data limitations within our data set resulted to unexplained variance, however, despite these limitations, the study demonstrates that historical museum samples provide a valuable data source that can fill research sampling gaps and improve our understanding of biodiversity patterns of the coral fauna in under sampled marine ecosystems. |
| **Order of Authors:** | ZOLEKA PATRICIA NONTLANTLA FILANDER, MSc |
| | Kerry Jennifer Sink |
| | Marcelo Visentini Kitahara |
| | Stephen Cairns, Doctorate |
| | Amanda L Lombard |
| **Additional Information:** | |
| **Question** | **Response** |
| **Financial Disclosure**<br><br>Enter a financial disclosure statement that describes the sources of funding for the work included in this submission. Review the submission guidelines for detailed requirements. View published research articles from *PLOS ONE* for specific | The financial support for this research study was provided by the Department of Forestry, Fisheries, and Environment (DFFE). |

*Powered by Editorial Manager® and ProduXion Manager® from Aries Systems Corporation*

| | |
|---|---|
| examples.<br><br>This statement is required for submission and **will appear in the published article** if the submission is accepted. Please make sure it is accurate.<br><br>**Unfunded studies**<br>Enter: *The author(s) received no specific funding for this work.*<br><br>**Funded studies**<br>Enter a statement with the following details:<br>• Initials of the authors who received each award<br>• Grant numbers awarded to each author<br>• The full name of each funder<br>• URL of each funder website<br>• Did the sponsors or funders play any role in the study design, data collection and analysis, decision to publish, or preparation of the manuscript?<br>• **NO** - Include this sentence at the end of your statement: *The funders had no role in study design, data collection and analysis, decision to publish, or preparation of the manuscript.*<br>• **YES** - Specify the role(s) played.<br><br>* typeset | |
| **Competing Interests**<br><br>Use the instructions below to enter a competing interest statement for this submission. On behalf of all authors, disclose any competing interests that could be perceived to bias this work—acknowledging all financial support and any other relevant financial or non-financial competing interests.<br><br>This statement is required for submission and **will appear in the published article** if the submission is accepted. Please make sure it is accurate and that any funding sources listed in your Funding Information later in the submission form are also declared in your Financial Disclosure statement. | The authors have declared that no competing interests exist. |

*Powered by Editorial Manager® and ProduXion Manager® from Aries Systems Corporation*

| | |
|---|---|
| View published research articles from [*PLOS ONE*] for specific examples.<br><br>**NO authors have competing interests**<br><br>Enter: *The authors have declared that no competing interests exist.*<br><br>**Authors with competing interests**<br><br>Enter competing interest details beginning with this statement:<br><br>*I have read the journal's policy and the authors of this manuscript have the following competing interests: [insert competing interests here]*<br><br>* typeset | |
| **Ethics Statement**<br><br>Enter an ethics statement for this submission. This statement is required if the study involved:<br><br>• Human participants<br>• Human specimens or tissue<br>• Vertebrate animals or cephalopods<br>• Vertebrate embryos or tissues<br>• Field research<br><br>Write "N/A" if the submission does not require an ethics statement.<br><br>General guidance is provided below. Consult the submission guidelines for detailed instructions. **Make sure that all information entered here is included in the Methods section of the manuscript.** | N/A |

| | |
|---|---|
| **Format for specific study types**<br><br>**Human Subject Research (involving human participants and/or tissue)**<br>• Give the name of the institutional review board or ethics committee that approved the study<br>• Include the approval number and/or a statement indicating approval of this research<br>• Indicate the form of consent obtained (written/oral) or the reason that consent was not obtained (e.g. the data were analyzed anonymously)<br><br>**Animal Research (involving vertebrate animals, embryos or tissues)**<br>• Provide the name of the Institutional Animal Care and Use Committee (IACUC) or other relevant ethics board that reviewed the study protocol, and indicate whether they approved this research or granted a formal waiver of ethical approval<br>• Include an approval number if one was obtained<br>• If the study involved *non-human primates*, add *additional details* about animal welfare and steps taken to ameliorate suffering<br>• If anesthesia, euthanasia, or any kind of animal sacrifice is part of the study, include briefly which substances and/or methods were applied<br><br>**Field Research**<br><br>Include the following details if this study involves the collection of plant, animal, or other materials from a natural setting:<br>• Field permit number<br>• Name of the institution or relevant body that granted permission | |
| **Data Availability**<br><br>Authors are required to make all data underlying the findings described fully available, without restriction, and from the time of publication. PLOS allows rare exceptions to address legal and ethical concerns. See the PLOS Data Policy and FAQ for detailed information. | Yes - all data are fully available without restriction |

| | |
|---|---|
| A Data Availability Statement describing where the data can be found is required at submission. Your answers to this question constitute the Data Availability Statement and **will be published in the article**, if accepted.<br><br>**Important:** Stating 'data available on request from the author' is not sufficient. If your data are only available upon request, select 'No' for the first question and explain your exceptional situation in the text box.<br><br>Do the authors confirm that all data underlying the findings described in their manuscript are fully available without restriction? | |
| **Describe where the data may be found in full sentences. If you are copying our sample text, replace any instances of XXX with the appropriate details.**<br><br>• If the data are **held or will be held in a public repository**, include URLs, accession numbers or DOIs. If this information will only be available after acceptance, indicate this by ticking the box below. For example: *All XXX files are available from the XXX database (accession number(s) XXX, XXX.).*<br>• If the data are all contained **within the manuscript and/or Supporting Information files**, enter the following: *All relevant data are within the manuscript and its Supporting Information files.*<br>• If neither of these applies but you are able to provide **details of access elsewhere**, with or without limitations, please do so. For example:<br><br>*Data cannot be shared publicly because of [XXX]. Data are available from the XXX Institutional Data Access / Ethics Committee (contact via XXX) for researchers who meet the criteria for access to confidential data.*<br><br>*The data underlying the results presented in the study are available from (include the name of the third party* | All relevant data are within the manuscript and its Supporting Information files. |

| | |
|---|---|
| *and contact information or URL).*<br>• This text is appropriate if the data are owned by a third party and authors do not have permission to share the data.<br><br>* typeset | |
| Additional data availability information: | Tick here if the URLs/accession numbers/DOIs will be available only after acceptance of the manuscript for publication so that we can ensure their inclusion before publication. |

Powered by Editorial Manager® and ProduXion Manager® from Aries Systems Corporation

Manuscript

Click here to access/download;Manuscript;Filander et al. 2023_PLOSone_manuscript text_.docx

# The application of historic sample-specific variables in evaluating the biodiversity patterns of the South African azooxanthellate scleractinians (Cnidaria: Anthozoa).

Zoleka N. Filander [1,2*], Kerry J. Sink [3,4], Marcelo V. Kitahara [5,6], Stephen D. Cairns [6], Amanda T. Lombard [4].

**1.** Biodiversity and Coastal Research, Oceans and Coasts, Department of Forestry, Fisheries, and Environment, Cape Town, South Africa. **2.** Zoology Department, Nelson Mandela University, Port Elizabeth, South Africa. **3.** South African National Biodiversity Institute, Cape Town, South Africa. **4.** Institute for Coastal and Marine Research, Nelson Mandela University, Port Elizabeth, South Africa**. 5.** Centro de Biologia Marinha, Universidade de São Paulo, São Sebastião, Brazil **6.** Department of Invertebrate Zoology, Smithsonian Institution, Washington DC, USA.

**Corresponding author** zfilander@gmail.com

## Abstract

Azooxanthellate scleractinian corals, a group of species that lack a symbiotic relationship with dinoflagellates, are influenced by environmental variables at various scales. As the global commitment to sustainably manage ocean ecosystems and resources rises, there is a growing need to describe biodiversity trends in previously unsampled areas. Benthic invertebrate research in South Africa is a developing field, and many taxa in deep water environments remain inadequately characterized. Recently, the South African azooxanthellate scleractinian fauna was taxonomically reviewed, but their distributional correlation with physical parameters have not been studied. Here we aim to understand the biodiversity gradients of the South African azooxanthellate coral fauna

by analysing the environmental correlates of museum samples. The associated coordinate data were georeferenced and depth obtained from a national bathymetric dataset, prior to undertaking a multivariate analysis. Overall, our results confirmed two longitudinal groups (eastern margin [group A] vs southern and western margin [group B]) and 11 depths represented within two bathymetric zones (shallow [50-200 m] and deep [300-1000 m]). Both the longitudinal groups and depth zones partially explained coral distribution patterns, with depth highly correlated with species variation. Caryophylliids, flabellids, and dendrophylliids contributed the most towards distinguishing longitudinal and depth gradients. Data limitations within our data set resulted to unexplained variance, however, despite these limitations, the study demonstrates that historical museum samples provide a valuable data source that can fill research sampling gaps and improve our understanding of biodiversity patterns of the coral fauna in under sampled marine ecosystems.

**Keywords:** stony Cold-water corals, longitude, depth, gradients, distribution.

## 1. Introduction

The distribution of azooxanthellate corals, a group of scleractinian species that lack a symbiotic relationship with photosynthetic dinoflagellates, is influenced by environmental variables at various scales (Guinotte et al., 2006; Hovland, 2008; Roberts et al., 2009; Davies and Guinotte, 2011; Angeletti et al., 2020). Physical and chemical oceanographic factors, as well as geomorphologic settings affect food supply and, consequently, benthopelagic coupling (Roberts et al., 2009). Overall, depth might be used as a variable linked to several oceanographic factors that influence species distributions. For example, coral species have preferred thermal ranges (Davies and Guinotte, 2011), and a global azooxanthellate coral richness trend has been

documented between 200 and 1000 m deep. This depth range often coincides with shelf and slope features, which may provide suitable substrate for larval settlement and habitats for azooxanthellate coral species to colonise (Cairns, 2007; Roberts et al., 2009). Furthermore, long-term environmental stability appears to also be important for the occurrence/distribution of deep water stony coral species. In addition to the temporal and spatial stability of an environment, it is well established that life history patterns, including reproduction strategies and relationship to substrate, are of utmost importance for a species' distribution (Oakham, 2009). For instance, attached deep water scleractinians require consolidated substrates to survive, whilst unattached forms are found on or in unconsolidated substrates (Roberts et al., 2009; Hovland, 2008).

Given the difficulty of sampling in deep-water marine systems, the mapping and classification of biodiversity into spatial units (which then act as surrogates for unmapped biodiversity) is a common approach in spatial planning (Waters, 2008; Costello, 2009; Reygondeau and Dunn, 2018; Reygondeau 2019; Richter et al., 2022). Considering the growing concern regarding declining ocean health, voluntary commitments to reach a national 30 % area protection by 2030, and the United Nations call for better ocean governance (United Nations, 2018; 2019; 2020), such spatial classifications are powerful tools to guide conservation and management strategies to support the achievement of the United Nations 14$^{th}$ Sustainable Development Goals (SDGs). Although ocean basins have been mapped at broad scales (Sayre et al., 2017) and several global and regional bioregionalisations exist (Ekman, 1953; Briggs, 1974; Spalding et al., 2007; Grant et al., 2006; Cedras et al., 2020; McQuaid et al., 2023), few studies have used species data to describe biological patterns in areas deeper than 200 m (Zezina, 1997; O'Hara et al., 2011; Watling et al., 2013; Cedras et al., 2020; Summers and Watling, 2021; Watling and Lapointe, 2022), particularly

within benthic ecosystems (O'Hara et al., 2011; Summers and Watling, 2021; Watling and Lapointe, 2022). Developing nations, such as South Africa, particularly lack specialised resources to survey deeper waters, further constraining *in situ* research (Bell et al., 2022). Consequently, samples housed in scientific collections may be a valuable source of biological data to evaluate distribution patterns (Spalding et al., 2007; Thandar et al., 1989; Woolley et al., 2020) but should be interpreted with caution.

Despite the fact that the first marine collections along South Africa's shores dates back to the 1700s (Day, 1977; Griffiths et al., 2010), ocean resource management is still constrained by the poor state of knowledge of key invertebrate species (Sink et al., 2019). Endeavouring to address such species data gaps, local research advancements have recently been initiated by examining natural history collections (Biccard 2012; Laird 2013; Filander, 2014; Olbers 2016; Boonzaaier, 2017; Landschoff 2011). Some of these studies have been integrated into the ecosystem map developed by Sink et al. (2019; 2023) for the National Biodiversity Assessment (NBA). The NBA used pelagic and benthic data, including biological available information (i.e., macrofauna, epifauna, and fish) to produce an expert-driven ecosystem type map for national reporting frameworks. It comprises four hierarchical levels that represent six ecoregions (two deep ocean and four confined to the continental shelf), five depth zones (shore, shelf, slope, plateau, and abyss), and different substrate types. Absent, however, from this national spatial classification map is a holistic consideration of the South African azooxanthellate scleractinian fauna, given that this taxonomic group was only recently reviewed (Filander et al., 2021), and its distribution patterns had not yet been investigated. The NBA does however report on some distribution records of

potential Vulnerable Marine Ecosystem indicator taxa, which includes records of two reef-building azooxanthellate coral taxa (i.e., Dendrophylliidae Gray, 1847 and Caryophylliidae Dana, 1846).

Earlier international studies (Cairns, 2007) have grouped the available literature on azooxanthellate Scleractinia into broad geographic regions. Although not a biodiversity analysis, Cairns (2007) produced an output that served as a starting point for emerging taxonomists in the field. Cairns and Keller (1993) did, nonetheless, summarise depth affiliations within the southwest Indian Ocean, in which South African taxa reported off the eastern and southern margins were represented. Apart from these two publications (Cairns and Keller, 1993; Cairns, 2007), the South African azooxanthellate Scleractinia distribution pattern have not been investigated in light of its relationship to physical variables. Therefore, this paper aims to examines the diversity measures of the South African azooxanthellate coral fauna, with respect to sample-specific environmental gradients.

## 2. Material and Methods

Data considered for this study were based primarily on a subset of species distribution records for the South African azooxanthellate scleractinian fauna recently reported by Filander et al. (2021). The subset was compiled by including those occurrence data with coordinate information and a sample number, resulting in 761 occurrence records (**Figure 1** and **Appendix A**: Occurrence data). These coral occurrence data were predominately collected during six historical dredge surveys undertaken between 1898 and 1990 (i.e., RV *Anton Bruun*, Benguela IV, RV *Meiring Naude*, RV *Pieter Faure*, Sardinops, and University of Cape Town Ecological Surveys). The recent surveys undertaken in the 21$^{st}$ century are represented by two trawl (NANSEN and Department of

Environment, Forestry and Fisheries/South African Environmental Observation Network demersal surveys) and three dredge research surveys (ACEP: Deep–Secrets and IMIDA surveys, Department of Environment, Forestry and Fisheries) ~~(see Filander et al., 2021).~~

**Figure 1**. Study domain and spatial coverage of the coral records forming the basis of the analysis.

It is worth noting that the historical data sets had a varying degree of reliability in terms of associated data and, therefore, required data validation. Consequently, all the occurrence records were first geo-referenced using ArcGIS 10.1. This step involved overlaying the coral point data on the National Biodiversity Assessment (Sink et al., 2019; 2023) marine ecosystem types map. Records that were recovered on the coastline were moved to the closest polygon boundary of the ecosystem types with the near command in ArcGIS 10.1. This process was particularly beneficial for the *Pieter Faure* stations, which had positions in degrees magnetic North (not true North); whereby land bearings were used as a reference. In the next step, the spatial join tool was used to assign depth in relation to the most recent national bathymetric dataset (de Wet and Compton, 2021) to each of the coral records, irrespective of whether depths were present in the coral archive data set or not. Depth contours started at 50 m and continued every 100 m isobath intervals to a maximum of 1000 m. The resulting data set consisted of 95 of the total 108 azooxanthellate scleractinian species known from South Africa (Filander et al., 2021).

## 2.1. Assumptions and sampling biases

Over 80% of the resulting data is of historical origin, and therefore poses some limitations. One of these limitations is sampling coverage bias, given that past national marine surveys focused mainly on nearshore areas due to their accessibility, whilst sampling in areas beyond the continental shelf

relied on international surveys (the *Pieter Faure* expeditions being an exception) (Griffiths et al., 2010). Nevertheless, these historical surveys represent decades of sampling effort but were not systematic and provide presence-only data. Secondly, depth and co-ordinate information are the only two variables commonly associated with such datasets, but may be unreliable in some instances (i.e., the *Pieter Faure* collection). Thus, the use of the occurrence data in the multi-variate analysis required three assumptions that may not necessarily be a true reflection of the data attributes. These include presenting the data as presence-absence, inferring the occurrence of the historical records to the modern day, and applying longitude and depth as a proxy for ocean basin and water mass properties; respectively. The above-mentioned two-part data geo-referencing methodology was therefore also undertaken to standardize the associated collection specific parameters for the application of longitude and depth data as abiotic variables.

Furthermore, the data preparation methodology does not follow the interpolation of the presence-absence matrix (i.e., if species occur between two extreme points, then occurrence is assumed in between) as conducted in preceding marine benthic invertebrate studies based on museum specimens (Filander, 2014; Olbers, 2016; Boonzaaier, 2017). This approach would have yielded unrealistic conclusions in the absence of fine-scale substrate data sources- as substrate type is one of the primary drivers of coral settlement. Whereas the 2018 NBA (Sink et al., 2019) sub-divided substrate into ecosystem types, the multibeam data represented less than 1% of the South African seabed. Cawthra et al. (2021) review on existing South African core samples collected from areas deeper that 130 m highlights the importance of high resolution hydroacoustic surveys to better contextualize published core localities. It is for this reason that the substrate level was not considered to support interpolation techniques.

Lastly, the average taxonomic distinctiveness (ATD) diversity measure was based on the established phylogenetic clades (Kitahara et al., 2010; Stolarski et al., 2011). However, owing to limited resolution regarding species relationships below family level, phylogenetic scores were not assigned to taxonomic levels lower than family. Additionally, the existing phylogenetic reconstructions still lack sufficient representation of azooxanthellate coral species occurring in South Africa. For instance, less than 20% of South African coral species have been sequenced and included in existing molecular trees (Kitahara et al., 2010; Stolarski et al., 2011). It is important to note that ATD is a diversity calculation method that considers the distance between each species and its closest relative outside the group. This calculation is then divided by the number of species within the group being evaluated. The resulting ATD value provides an estimation of the group's evolutionary uniqueness, with higher values indicating greater distinctiveness. Consequently, an alphabetically arranged method would yield inaccurate results.

**2.2. Data analysis**

A presence-absence matrix (**Appendix A**: presence-absence) of the coral occurrence data was compiled and all analyses were undertaken using the PRIMER 7 software package (Clarke and Warwick, 2001; Clarke et al., 2014), with the PERMANOVA+ add on (Anderson et al., 2008). The matrix, consisting of 488 columns (stations/samples) and 95 rows (species), was converted to a resemblance matrix. The associated higher taxonomic classifications of these resulting species identifications were thereafter extracted from the World Register of Marine Species batch match online function (WoRMS Editorial Board, 2021) (**Appendix A**: Taxonomic attributes). Owing to the patchy nature of the data set, in which 30 species were represented by only one sample and 22 species by less than ten samples (**Appendix A**: Number of records per species), the Gamma+

dissimilarity matrix was selected (Clark and Warwick, 1999; Clark et al., 2006). This measure used the cophenetic distances derived from the phylogeny established in Kitahara et al. (2010) and Stolarski et al. (2011) (e.g. “Basal”, “Complex”, and “Robust”) (**Appendix A**: Taxonomic attributes). Such a procedure allowed for biotic distances among samples to be quantified even when they had zero or very few species in common.

The sample-specific data also required data preparation, which followed the biological data assessment. Longitude and depth are the two sample-specific variables considered to determine the environmental settings of the South African maritime domain (**Appendix A**: Sample-specific abiotic data). For instance if a sample was recorded at a 31° longitude, then it was collected in the Indian Ocean and influenced by the Agulhas Current. Additionally, water mass temperature (T) and salinity (S) properties can be confirmed by depth, in which upper layer waters were identified between the surface and 500 m deep, intermediate between 500 and 1500 m, and abyss greater that 1500 m (Emery, 2003). Each abiotic parameter was firstly classified accordingly (see below), prior to running an one-way (unordered) similarity analysis (ANOSIM) test to evaluate variation of corals species within the longitudinal and depth groups (**Appendix A**: Sample-specific abiotic data). The ANOSIM test requires groupings and measures the mean of ranked dissimilarities between groups to the mean of ranked dissimilarities within groups. A standard approach was undertaken to investigate change in species attributes along the longitudinal and depth gradient, whereby a SIMPER analysis was run to evaluate contributing taxa.

To classify the longitudinal data as a factor to be tested on the biological data, an auto select k-R cluster mean analysis was run on the normalized longitudinal data. A draftsman’s plot was

produced to identify the number of longitudinal groups present and validate the cluster groups present (**Appendix B**: Figure 2). The depth classification starts at a 50 m isobath and progresses at 100 m increments. These depth readings were further classified according to zone, i.e., shallow (50-200 m) *vs* deep (300-1000 m). The depth values and subsequent depth zones were also added as a factor for testing. Similarity percentage (SIMPER) tests were run independently to distinguish coral species contributing to the longitudinal groups identified by the k-R mean analysis and depth groups. Sampling effort (i.e., denoted by N), species richness (denoted by S), Shannon index (denoted by $H'log^{e}$), and taxonomic distinctiveness (denoted by delta+) across the longitudinal and depth groups was quantified. The former was investigated by assigning coral records to 50x50 km grids created with the fishnet ArcGis function, whereby the grid size was guided by the boundary breaks of the k-R mean cluster groups.

Subsequently, a RELATE routine was undertaken to evaluate if the combined longitude and depth spatial gradients correspond with those inferred from the coral species patterns. Here we used the Gamma + matrix in relation to the associated depth and longitude information, which was normalised into a Euclidean distance resemblance matrix. The RELATE technique calculated a Spearman's $\rho$ rank correlation coefficient between all elements of the coral assemblage and environmental variable resemblance matrices, followed by a permutation test. Following this, a biota and/or environment matching (BEST) test was conducted to confirm which variable contributed the most to sample statistic given by the RELATE results. A species accumulation model was lastly produced to assess how well the current observed azooxanthellate stony coral data represents South Africa's predicted coral diversity.

# 3. Results

## 3.1. Longitudinal gradient

The k-R (non-hierarchal) cluster analysis yielded two longitudinal groups (R = 0.94), whereby group A encompasses samples from the eastern margin of South Africa and group B are samples from the southern and western margins (**Figure 3**). The SIMPER results suggested that biological samples represented by each of the two longitudinal clusters had an overall low average similarity in species composition (**Appendix C**: Long group SIMPER spp results). Communities in group A, with an average similarity of 3.4 %, were characterised by a total of 11 species from five families (i.e., Dendrophylliidae, Caryophylliidae, Micrabaciidae Vaughan, 1905, Turbinoliidae Milne-Edwards & Haime, 1848, and Flabellidae Bourne, 1905). Group B had a total of eight species contributing to the group identity with a slightly higher average similarity (4.6%), whereby only two families (i.e., Dendrophylliidae and Caryophylliidae) were represented.

**Figure 3**. The 50x50 km gridded cells with samples in relation to the longitudinal groups defined by the k-R cluster analysis. Group A represents samples collected off the eastern margin and group B are samples collected off the southern and western margins.

Furthermore, there was a distinction between families contributing the most to the cluster identities. Three dendrophylliids (*Balanophyllia capensis* Verrill, 1865), *Pourtalopsammia togata* (van der Horst, 1927), and *Heteropsammia cochlea* (Spengler, 1781) contributed the most to the similarity within group A; and the caryophylliids (*Trochocyathus* sp. 2 sensu Filander et al. (2021), *Caryophyllia stellula* Cairns, 1998, and *Desmophyllum dianthus* (Esper, 1794)) defined group B. When comparing the two groups, a 98% average dissimilarity was observed when investigating species contributing to a minimum cut off of 70%, whereby different species influenced group

distinction. *Heteropsammia cochlea*, *Letepsammia franki* (Owens, 1994), *Flabellum pavoninum* (Lesson, 1831), and *Labyrinthocyathus delicatus* (Marenzeller, 1904) were represented only in group A. Among these species, *L. delicatus* is restricted to the Indian Ocean, whilst *L. franki*, *F. pavoninum* and *H. cochlea* are widely distributed in the Indo-Pacific. Four species contributed the most to group B, of which two are restricted to South African waters (*Ednapsammia columnapravia* Filander, 2021 and *Dendrophyllia* sp. 1 sensu Filander et al. (2021)), one considered cosmopolitan (*Desmophyllum pertusum* (Linnaeus, 1758)), and the remaining reported from the Atlantic and Indian ocean basins (*Enallopsammia pusilla* (Alcock, 1902)) (**Appendix C**: Long Group SIMPER spp results).

Overall, the number of samples between the two groups varied, whereby group A (eastern margin) had over two times more samples than group B (southern and eastern margin) (**Table 1**). Contrary to this, the related area (i.e., number of grids) representing these samples was larger in group B than in group A (**Table 1**). Diversity followed the same pattern of higher measures in group A as compared with group B.

**Table 1**. Summary of sampling effort in relation to longitudinal gradient

| k-R cluster group | Number of samples | Number of 50x50 km grids | Species richness | Shannon's Index | Delta + |
|---|---|---|---|---|---|
| **A** (eastern margin) | 569 | 37 | 86 | 3.964 | 90.907 |
| **B (**southern & western margin) | 192 | 43 | 37 | 3.249 | 89.289 |

## 3.2. Depth gradients

A direct relationship between the number of samples (N), species richness (S), and depth was observed (**Figure 4**). The highest number of samples and observed species richness occurred between depths of 50 and 200 m, with the greatest species richness and sample count recorded at a depth of 50 m. The same two measures (S and N) fluctuated at the deep bathymetric zone (i.e., 300-1000 m). Within this deep zone, the highest coral diversity measures (S and N) were recorded at 1000 m and the lowest at 800 m. The overall pattern suggests that species richness is influenced by depth and sampling effort, but other factors may also play a role in shaping patterns of species diversity across different depths. Average taxonomic distinctiveness (denoted by delta +), which takes into account species phylogeny, did not show a clear pattern in coral diversity with depth and species diversity was relatively constant from 50 to 200 m. However, according to this measure, coral diversity was slightly higher at 1000 m despite the usage of a smaller number of samples from this depth (42 samples compared to 269 samples at 50 m). In other words, eight taxonomic families were recorded at 1000 m, while only seven were recorded at 50 m. In contrast, however, the conservative Shannon diversity index mirrored the pattern of species richness with depth (**Figure 4**).

**Figure 4**. The relationship between number of coral samples (N) and species richness (S) in conjunction with the average taxonomic distinctiveness (delta+) and Shannon diversity (H’log$^e$) index measures across depth gradients. The x-axis shows samples represented in depth values in metres and the y-axis shows values that represent diversity measures in arbitrary units. The inset shows the Shannon diversity (H’log$^e$) index repeated on a Y axis of 1-4.

The SIMPER results of the coral species data according to family suggested that the caryophylliids, dendrophylliids, and flabellids were the main contributing taxa towards both the shallow (50-200 m) and deep (300-1000m) stations. Whilst all three families collectively contributed towards the

zone comparison (i.e., shallow *vs* deep) at a 70% cut, the Caryophylliidae representatives were more abundant in the deep stations compared with the Dendrophylliidae and the Flabellidae in the shallow stations (**Appendix C**: Depth zones SIMPER family results).

When comparing the two bathymetric zones at lower taxonomical rankings, three species were exclusively recorded in the shallower zone (*Rhizopsammia compacta* Sheppard & Sheppard, 1991, *Truncatoflabellum inconstans* (Marenzeller, 1904), and *Dendrophyllia cornigera* (Lamarck, 1816)), whereas the deeper zones were characterized by three of the four species restricted to the longitudinal group B: *E. columnapravia, Dendrophyllia* sp. 1, and *E. pusilla.*

### 3.2. The correlation of sample-specific variables (longitude and depth groups) to coral distribution patterns

The one-way ANOSIM results, which evaluated the rank differences in the coral pattern that may be explained by the longitudinal k-R clusters and depth groupings independently, showed that the two longitudinal clusters (R=0.05, p = 0.001), and the eleven depth groupings (R = 0.072, p = 0.001) of the biological assemblages differed significantly from one another (**Appendix D**: Figure 5). Depth also showed significant differences (R = 0.105, p = 0.001) when grouped as shallow (50-200 m) and deep zones (300-500 m) (**Appendix D**: Figure 6). Nonetheless, even though some overlap was observed in the coral structure when testing longitude and depth; the null hypothesis (of no differences in rank groups) can be rejected.

The RELATE results showed a marginal correlation (Rho-value = 0.087) but a significant difference (p-value = 0.001) when comparing the coral patterns modelled by the Gamma+

resemblance matrix to that of the Euclidean distance matrix (i.e., environmental variables - longitude and depth; **Figure 7**). It is important to note that the null hypothesis in the RELATE function is that there is no correlation. Thus, although the correlation is closer to zero (unexplained variance), the p-value confirms that longitude and depth are good predictors for the coral distribution patterns. The BEST results further confirmed that depth had an independent correlation value of 0.094, whilst both environmental parameters (longitude and depth) accounted for a correlation value of 0.097.

**Figure 7**. Simulated distribution/histogram of the test statistic Rho under the null hypothesis that there is no correlation between the modelled coral patterns and that of the environmental variables Rho = 0.041.

The majority of the species accumulation curves, which show how the number of species detected (i.e., observed or sampled) increases with increasing sampling effort (i.e., the number of individuals or samples collected), did not reach a plateau (**Figure 8**). All seven estimated curves, along with the observed or sampled species, started with a steep slope and indicated a rapid increase in the number of species observed with increasing sampling effort. Only two (MM and UGE) of the seven estimator curves followed the species observed pattern (Sobs), which appears to be levelling off as the sampling effort increases (**Figure 8**).

**Figure 8**. Species richness accumulation curve showing the species observed (Sobs= blue upright triangle) in relation to five estimators (Chao 1= red downward triangle, Chao 2= green square, Jacknife 1= pink diamond, Jacknife 2= blue circle, Bootstrap= grey cross). Two pairs of curves overlap, whereby the UGE estimator curve follows the same pattern as the Sobs and the Chao1 has the same pattern as Chao 2.

## 4. DISCUSSION

The multivariate analyses suggests that the sample-specific associated data (e.g., longitude and depth) are significant predictor variables for azooxanthellate Scleractinia coral diversity. Nonetheless, unexplained variance exists. Diversity measures were assessed, in which the number of samples showed an inversely proportional relationship with species richness. Contrary to this observation, taxonomic distinctiveness (a diversity measure independent of the number of samples) revealed an opposing pattern to that of the univariate Shannon index measure. Thus, taxonomic distinctiveness accounted for the uneven species distribution across the South African continental maritime domain.

An increasing species turnover along the west to east gradient was observed in our analysis. Such distributional patterns have long been reported for other South African marine invertebrates (e.g., Lang, 2012; Filander, 2014; Boonzaaier, 2017), suggesting that different oceanographic conditions are influencing the South African marine fauna. The accompanying current regimes may also govern these contrasting species profiles across the region. Thus, although the two longitudinal boundaries (Group A = eastern margin *vs* Group B= western margin) established by the k-R mean cluster analysis do not conform to the previously proposed oceanographic boundaries (Longhurst, 2007; Spalding et al., 2012;), the ANOSIM suggested a species pattern that may be explained by the two longitudinal groups. These margins correspond to varying oceanographic variables and currents, whereby the eastern margin (group A) is situated within the oligotrophic waters of the Indian oceanic basin and influenced by the western boundary Agulhas current. Interestingly, group B encompasses the southern and western margins located in both the Indian and Atlantic basins respectively. At the southern margin, the Agulhas current retroflects, moving away from the shelf,

and introduces Indo-Pacific waters into the Atlantic Ocean, the latter being regulated by the northward flowing Benguela current (Shannon, 1985).

The SIMPER results detailed a clear taxonomic/ family and species distinctions within these two longitudinal groups. Dendrophylliids contributed the most to Group A samples and caryophylliids to Group B. Additionally, the exclusivity in species found between Group A (*H. cochlea*, *L. franki*, *F. pavinonum*, and *L. delicatus*) and Group B (*E. columnapravia*, *Dendrophyllia* sp. 1, *D. pertusum*, and *E. pusilla*) (see longitudinal gradient results) corroborates with the proposal that species have a temperature threshold (Roberts et al., 2009; Cairns, 2007). The physiological characteristics of azooxanthellate coral species are indeed influenced by the properties of ambient water temperature (Gori et al., 2016; Castellan et al., 2019). For example, an *ex-situ* experiment undertaken on the reef-building corals *D. pertusum* and *Madropora oculata* revealed that they respond differently when exposed to three temperatures (12, 9.0, and 6.0 °C; Naumann et al., 2014). The respiration response rates varied; *M. oculata* declined whereas *D. pertusum* was not affected by temperatures being lowered. Two other physiological responses (i.e., calcification and dissolved organic carbon) were measured, and neither showed a consistent trend when comparing the two species. Thus, species belonging to different families or even congeners are expected to exhibit varying thermal tolerance.

The recovered species longitudinal pattern of low sampling effort in Group A (eastern margin) but higher number of records and diversity observed herein, was particularly surprising as the western margin (which contributes to Group B) has a higher historic sampling effort (Griffiths et al., 2010). The greater presence of coral species in the eastern Agulhas region (Group A) may be explained

by the heterogenous seabed substrate types provided by the increased abundance of mesophotic reefs, submarine canyons, and mosaic ecosystem types (Sink et al., 2019). Whilst the incising submarine canyons along the eastern continental margin (Green et al., 2007; Green, 2008; 2009; Green, 2011) may also give rise to a heterogenous environment, localised canyon substrate type studies need to be undertaken to confirm such hypothesis (Filander et al., 2022). Even though the Benguela Current in the South Atlantic (influencing the western passive margin) is unique in its interactions with the western boundary Agulhas current (Longhurst, 2007), this region has substrate predominately unconsolidated, resulting in a more homogenous environment (Dingle,1979; Cawthra et al., 2021; Filander et al., 2022). Additionally, dissolved oxygen levels have been proposed to affect scleractinian growth (Hanz et al., 2019) and the Southern Benguela Upwelling region does include a low-oxygen area/cell off St Helena Bay (Lamont et al., 2015). Though nearshore, such cells are reported to show spatial variability and may modify offshore upwelling water masses. These oxygen parameters superimposed with unconsolidated bottom types and a slow current (< 3 m/s) may be a constraint for coral presence. The presence of coral is however influenced by multiple factors operating at different scales, and it is crucial to consider species-specific regional adaptation abilities to environmental gradients (i.e., dissolved oxygen) - even for cosmopolitan species (Orejas et al., 2021). Nonetheless, the prominence of anthropogenic activities that interact with the seabed in the Southern Benguela Upwelling area (Atkinson et al., 2011; Majiedt et al., 2019) cannot be overlooked and may also influence the low number of species records in the area.

The southern margin, which contributes to Group B, is a unique area that exhibits minimal interaction with other landmasses and, as such, high endemism has been noted (Griffiths et al.,

2010). In this region, the Agulhas current injects Indo-Pacific waters into the Atlantic, down to depths of 2000 m in the form of anticyclonic rings (Beal et al., 2011), before retroflecting eastwards towards the Southern Indian Ocean Gyre and the Antarctic circumpolar current (Spalding et al., 2012). Schouten et al. (2000) noted that the location of the retroflection loop is variable, but still within the southern region. Nonetheless, the Agulhas transport is estimated to increase from 65 Sv (1Sv - $10^{6}$ m $^{3}s^{-1}$) at 32°S to 95 Sv at the southern tip of South Africa, as it breaks away from the shelf (Gordon et al., 1992; Duncombe Rae, 1991). Thus, the unpredictable behaviour and velocity of the Agulhas current make this area challenging for sampling and, therefore, the low number of records here may be attributed to limited sampling effort.

The analysis of depth gradients allowed patterns of species richness in relation to the depth to be better understood. These results complement the longitudinal gradients whereby the univariant biodiversity measures peaked at 50 m, which corresponds to the accessible eastern margin of the South African maritime domain. In addition to the shelf being shallower (~ 50-150 m) and more accessible, the western boundary Agulhas current (characteristic of this area) has been linked to the highly diverse biological properties in the Southwest Indian Ocean, where eddies can trap and transport material over long distances (Halo et al., 2014). These complex oceanographic eddies can upwell deep nutrient-rich waters through surface divergence mechanisms (Halo et al., 2014), creating environments that favour the continuous inflow of potential food sources. Thus, these observations may provide grounds for a hypothesis to explain why azooxanthellate corals have a higher presence within this area.

The multivariate taxonomic average distinctiveness measure (denoted by delta +) showed diversity to be highest at 1000 m, in which eight of the eleven known South African coral families are represented. This result aligns with the knowledge that the global azooxanthellate stony coral pattern (Cairns, 2007) has overall higher species diversity between the 200 and 1000 m. Irrespective, the SIMPER analysis distinguished three major families to contribute to bathymetric zone delineation. The deeper depths (300-1000 m) were characterized by caryophylliids and flabellids, and the shallow zone (50-200 m) by dendrophylliids. These results conforms with the known depth affiliations of these families, in which Dendrophylliidae species occurrence is reported to peak at shallower depths (50 to 300 m) (Cairns, 2001) and extant species of Caryophylliidae and Flabellidae are more prominent in the deeper waters (more than 200 m) (Kitahara, 2011).

The two sample-specific (i.e., depth and longitude) data sets were applied in combination to extrapolate ocean basin properties (nutrient content, salinity, temperature, etc.), which characterise the oceanographic settings influencing South African marine fauna (i.e., the colder Benguela current along the western margin, and the warmer Agulhas current along the southern and eastern margin). In this context, the permutation models (ANOSIM and RELATE) imply that longitude and depth are good predictors for coral distribution patterns. However, the close to zero R-values ($R<0.5$) suggests a non-linear relationship even though significant variability is evident in the species composition within the factorial groups. Whilst depth is noted to be one of the main drivers for coral distribution (as shown by BEST results), it is important to recognize that this parameter encompasses several other properties, such as the Aragonite Saturation Horizon (ASH) that is the depth below which calcium carbonate becomes unstable and tends to dissolve (Jiang et al., 2015;

Guinotte et al., 2006). Such a zone has been estimated at 700-1500 m depth range south of ~ 20°S (Jiang et al., 2015). Eight of the eleven known South African coral families are recorded within this depth range, suggesting these species are surviving within an aragonite saturation state. Interestingly, coral species have been previously reported to withstand saturating conditions. For example, a study undertaken in the Caribbean basin showed the depth of the aragonite saturation horizon to be strongly related to coral assemblage variation, whereby *M. oculata* and *S. variabilis* occur in patchy distributions at or above the saturation zone (Auscavitch et al., 2020). The response of coral species to water properties, such as the ASH, are in no way consistent, highlighting the need for further research to comprehend the underlying environmental drivers of coral distribution.

Although the azooxanthellate coral data reported herein represent an accumulation of samples over 30 years and are the best available representation of the South African fauna, all species richness estimator models did not plateau, demonstrating that the area is still not well sampled and may be much more diverse than currently known. The shape and slope of the curve typically provides information on the species richness, evenness, and heterogeneity of the community being sampled, as well as the adequacy of the sampling effort. In other words, if the species accumulation curve keeps increasing with additional sampling effort then full extent of species diversity within the study area has not yet been captured. Without a doubt, additional sampling coverage will provide clearer conclusions on national coral diversity trends.

## 4. Conclusion and recommendations

This study examined the best available data for the South African azooxanthellate coral fauna and presented a pre-processing methodology that caters for historical samples. Differences in

azooxanthellate coral species across South Africa’s diverse and dynamic oceanographic conditions were revealed, whereby species turnover increased on a west to east axis. A species depth gradient was additionally observed, in which the multivariate diversity measure complemented the existing knowledge on taxa trends. These patterns were evident despite the data limitations related to museum samples. Whilst the lack of museum associated abiotic data still exists, the methodology to standardize co-ordinate information and depth may be considered in other data sets with similar attributes to inform further research to elucidate diversity patterns. In general, historical collections (which represent years of sampling effort) provide a valuable biological data source but require thorough validation. Despite the sparsity and unbalanced nature of the data, knowledge has been advanced and sampling gaps identified. A purposeful application for this existing coral data set will be its integration into multi-taxa biogeography analyses that will support ecosystem description and delineation. The data set will also be valuable for spatial prioritisation and marine spatial planning, particularly alongside taxa that share similar abiotic requirements.

## Acknowledgements

A sincere acknowledgement goes to Dr David Herbert (Department of Natural Sciences, National Museum Wales) who assisted with associated station data for the *Meiring Naude* and *Sardinops* collections. Dr Carl Palmer (Alliance of Collaboration in Climate and Earth System Science) is thanked for proof-reading the first manuscript draft, and Dr Victoria Goodall (Nelson Mandela University) for reviewing the data analysis section. Mr Ashley Johnson and Dr Lauren Williams (Department of Fisheries, Forestry, and the Environment) provided words of encouragement and ArcGIS technical support; respectively.

**References**

1. Alcock A. Diagnoses and descriptions of new species of corals from the "Siboga-Expedition".Tijdschrift der Nederlandsche Dierkundige Vereeniging. 1902; 7(2): 89–115.

2. Angeletti L, Castellan G, Montagna P, Remia A, Taviani M. The "Corsica Channel Cold-Water Coral Province"(Mediterranean Sea). Front Mar Sci. 2020; p.661. https://doi.org/10.3389/fmars.2020.00661

3. Anderson MJ, Gorley RN, Clarke KR. PERMANOVA+ for PRIMER: guide to software and statistical methods. Plymouth, UK: PRIMER-E; 2008.

4. Atkinson LJ, Field JG, Hutchings L. Effects of demersal trawling along the west coast of southern Africa: multivariate analysis of benthic assemblages. Mar Ecol Prog Ser. 2011; 430, 241–255. doi: 10.3354/meps08956

5. Auscavitch SR, Lunden JJ, Barkman A, Quattrini AM, Demopoulos AW, Cordes EE. Distribution of deep-water scleractinian and stylasterid corals across abiotic environmental gradients on three seamounts in the Anegada Passage. PeerJ. 2020; 8, p.e9523.

6. Beal L, De Ruijter W, Biastoch A, Zahn R, SCOR/WCRP/IAPSO Working group. On the role of the Agulhas system in ocean circulation and climate. Nature. 2011 ; 472, 429–436. https://doi.org/10.1038/nature09983

7. Bell KLC, Quinzin MC, Poulton S, Hope A, Amon, D. (Eds.). Global Deep-Sea Capacity Assessment. Ocean Discovery League, Saunderstown, USA; 2022. http://doi.org/10.21428/cbd17b20.48af7fcb

8. Biccard A. Taxonomy, systematics and biogeography of South African Cirripedia (Thoracica). M.Sc. Thesis, University of Cape Town. 2012. Available from: http://hdl.handle.net/11427/10163

9. Boonzaaier MK. Diversity and Zoogeography of South African Bryozoa. Doctoral thesis, University of Western Cape. 2017. Available from: http://hdl.handle.net/11394/6308

10. Bourne GC. Report on the solitary corals collected by Professor Herdman, at Ceylon, in 1902. Report to the Government of Ceylon on the Pearl Oyster Fisheries of the Gulf of Manaar. 1905; 4: 187-241, pls. 1-4.

11. Briggs JC. Operation of zoogeographic barriers. Syst Biol. 1974 Jun 1;23(2):248-56.

12. Cairns S. Azooxanthellate Scleractinia (Cnidaria: Anthozoa) of western Australia. Records of the Western Australian Museum. 1998; 18: 361–417. https://repository.si.edu/handle/10088/2465

13. Cairns SD. Deep-water corals: an overview with special reference to diversity and distribution of deep-water scleractinian corals. Bull Mar Sci. 2007 Nov 1;81(3):311-22.https://repository.si.edu/handle/10088/7536

14. Cairns S. A generic revision and phylogenetic analysis of the Dendrophylliidae (Cnidaria: Scleractinia). 2001. https://doi.org/10.5479/si.00810282.615

15. Cairns S, Keller N. New taxa distributional records of azooxanthellate Scleractinia (Cnidaria, Anthozoa) from the tropical southwest Indian Ocean, with comments on their zoogeography and ecology. Ann S Afr. Mus. 1993; 103, 213–292.

16. Castellan G, Angeletti L, Taviani M, Montagna P. The yellow coral Dendrophyllia cornigera in a warming ocean. Front Mar Sci 2019; 6, p.692.

17. Cawthra HC, Bergh EW, Wiles EA, Compton JS. Late Quaternary deep marine sediment records off southern Africa. S Afr J Geol. 2021; 124(4), pp.1007-1032.

18. Cedras R, Halo I, Gibbons M. Biogeography of pelagic calanoid copepods in the Western Indian Ocean. Deep Sea Res. 2020; 179, 104740. https://doi.org/10.1016/j.dsr2.2020.104740

19. Clarke K, Warwick R. The taxonomic distinctness measure of biodiversity: weighting of step lengths between hierarchical levels. Mar Ecol Prog Ser. 1999; 184, pp.21-29.

20. Clarke K, Somerfield P, Chapman M. On resemblance measures for ecological studies, including taxonomic dissimilarities and a zero-adjusted Bray–Curtis coefficient for denuded assemblages. J Exp Mar BioL and Ecology. 2006; 330(1), pp.55-80.

21. Clarke K, Gorley R, Somerfield P, Warwick R. Change in marine communities: an approach to statistical analysis and interpretation. Primer-E Ltd. 2014.

22. Costello M. Distinguishing marine habitat classification concepts for ecological data management. Mar Ecol Prog Ser. 2009; 397, 253–268. https://doi.org/10.3354/meps08317

23. Dana J. Zoophytes. Volume VII of the United States Exploring Expedition during the years 1838, 1839, 1840, 1841, 1842, under the command of Charles Wilkes, USN. Lea & Blanchard, Philadelphia, 740 pp. 1846. Available from: https://doi.org/10.5962/bhl.title.70845

24. Davies A, Guinotte J. Global habitat suitability for framework-forming cold-water corals. PloS one. 2011; 6(4), p.e18483. https://doi.org/10.1371/journal.pone.0018483

25. Day JH. Marine biology in South Africa. In: Brown AC, editor. A history of scientific endeavour in South Africa. Cape Town: Royal Society of South Africa. pp. 86–108. 1997

26. de Wet WM, Compton JS. Bathymetry of the South African continental shelf. Geo-Marine Letters. 2021 Sep;41(3):40.

27. Dingle RV.Sedimentary basins and basement structures on the continental margin of southern Africa. Bull Geol Surv S Afr. 1979; 63, 29–43.

28. Duncombe Rae C. Agulhas retroflection rings in the South Atlantic Ocean: an overview. S Afri J Mar Sci. 1991; 11(1), pp.327-344. https://doi.org/10.2989/025776191784287574

29. Ekman S. Zoogeography of the sea: Sidgwick and Jackson. Ltd, London. 1953.

30. Emery WJ. Water types and water masses. Encyclopedia of ocean sciences. 2001; 6, pp.3179-3187.

31. Esper E. Fortsetzungen der Pflanzenthiere. vol. 1, parts 1–2. Nürnberg, 64 pp. 1794.

32. Filander Z. Systematics and biodiversity of South African sea urchins. M.Sc. Thesis University of Cape Town. 2014.

33. Filander Z, Kitahara M, Cairns S, Sink K, Lombard A. Azooxanthellate Scleractinia (Cnidaria, Anthozoa) from South Africa.ZooKeys. 2021. https://doi.org/10.3897/zookeys.1066.69697

34. Filander Z, Smith AN, Cawthra HC, Lamont T. Benthic species patterns in and around the Cape Canyon: A large submarine canyon off the western passive margin of South Africa. Front Mar Sci. 2022; 9. https://doi.org/10.3389/fmars.2022.1025113

35. Gordon A, Weiss R, Smethie Jr W, Warner M. Thermocline and intermediate water communication between the South Atlantic and Indian Oceans. J Geophys Res. 1992; 97(C5), pp.7223-7240. https://doi.org/10.1029/92JC00485

36. Gori A, Ferrier-Pagès C, Hennige SJ, Murray F, Rottier C, Wicks LC, Roberts JM. Physiological response of the cold-water coral Desmophyllum dianthus to thermal stress and ocean acidification. PeerJ. 2016; 4:e1606https://doi.org/10.7717/peerj.1606

37. Grant S, Constable A, Raymond B, Doust S. Bioregionalisation of the Southern Ocean. Report of an expert workshop, Hobart, Australia, September 2006. World Wildlife Fund–Australia and Antartic Climate and Ecosystems Cooperative Research Centre. 2006.

38. Gray J.. An outline of an arrangement of stony corals. Ann Mag Nat Hist. 1847; (1)19: 120–128. https://doi.org/10.1080/037454809496460

39. Green AN, Goff JA, Uken R. Geomorphological evidence for upslope canyon-forming processes on the northern KwaZulu-Natal shelf, SW Indian Ocean, South Africa. Geo-Mar Lett. 2007; 27(6), pp.399-409.

40. Green A, Uken, R. Submarine landsliding and canyon evolution on the northern KwaZulu-Natal continental shelf, South Africa, SW Indian Ocean. Mar Geol. 2008; 254(3-4), pp.152-170.

41. Green A. Sediment dynamics on the narrow, canyon-incised and current-swept shelf of the northern KwaZulu-Natal continental shelf, South Africa. Geo-Mar Lett. 2009; 29(4), pp.201-219.

42. Green A. Submarine canyons associated with alternating sediment starvation and shelf-edge wedge development: Northern KwaZulu-Natal continental margin, South Africa. Mar Geol. 2011; 284(1-4), pp.114-126.

43. Griffiths C, Robinson T, Lange L, Mead A. Marine biodiversity in South Africa: an evaluation of current states of knowledge. PloS one. 2010; 5(8), p.e12008.

44. Guinotte J, Orr J, Cairns S, Freiwald A, Morgan L, George R. Will human- induced changes in seawater chemistry alter the distribution of deep- sea scleractinian corals?. Front Ecol Environ. 2006; 4(3), pp.141-146. https://doi.org/10.1890/1540-9295(2006)004[0141:WHCISC]2.0.CO;2

45. Halo I, Penven P, Backeberg B, Ansorge I, Shillington F, Roman R. Mesoscale eddy variability in the southern extension of the East M Madagascar Current: Seasonal cycle, energy conversion terms, and eddy mean properties. J Geophys Res. 2014; 119(10), pp.7324-7356.

46. Hanz U, Wienberg C, Hebbeln D, Duineveld G, Lavaleye M, Juva K, Dullo WC, Freiwald A, Tamborrino L, Reichart G.J, Flögel, S. Environmental factors influencing benthic communities in the oxygen minimum zones on the Angolan and Namibian margins. Biogeosciences. 2019; 16(22), pp.4337-4356.

47. Horst van der C. Eupsammid corals from South Africa. Union of South Africa Fisheries and Marine Biological Survey Report 5 (special reports). 1927; 1, 2: 1–7.

48. Hovland M.. Deep-water coral reefs: Unique biodiversity hot-spots Springer Science & Business Media. 2008.

49. Jiang L, Feely R, Carter B, Greeley D, Gledhill D, Arzayus K. Climatological distribution of aragonite saturation state in the global oceans. Glob Biogeochem Cycles. 2015; 29(10), pp.1656-1673. https://doi.org/10.1002/2015GB005198

50. Kitahara VM. Morphological and molecular systematics of scleractinian corals (Cnidaria, Anthozoa), with emphasis on deep-water species. PhD. Thesis.. James Cook University. 2011.

51. Laird M. Taxonomy, systematics and biogeography of South African actiniaria and corallimorpharian. PhD. Thesis. University of Cape Town, South Africa, 236 pp. 2013. Availanle from: http://hdl.handle.net/11427/6117

52. Lamarck J.-B. M. de. Histoire naturelle des animaux sans vertèbres. Tome second. Paris: Verdière, 568 pp. 1816. Available online at http://www.biodiversitylibrary.org/item/47698

53. Lamont T, Hutchings L, van den Berg MA, Goschen WS, Barlow RG. Hydrographic variability in the St. Helena B ay region of the southern Benguela ecosystem. J Geophys Res. 2015; 120(4), pp.2920-2944.

54. Landschoff J. Contributions to the taxonomy of South African hermit crabs (Crustacea: Decapoda: Paguroidea) – integrating microCT scanning and barcoding. PhD. Thesis. University of Cape Town, South Africa, 242 pp. 2018. Available from: http://hdl.handle.net/11427/28431

55. Lange L. Use of demersal bycatch data to determine the distribution of soft-bottom assemblages off the West and south coasts of South Africa. PhD. Thesis. University of Cape Town). 2012. Available from: http://hdl.handle.net/11427/10899

56. Lesson R.Illustrations de Zoologie. Arthus Bertrand, Paris, 3 pp. 1831. Available from: http://www.biodiversitylibrary.org/item/91260

57. Linnaeus C. Systema Naturae per Regna Tria Naturae, Secundum Classes, Ordines, Genera, Species, Cum Characteribus, Differentiis, Synonymis, Locis. Edition Decima, Reformata. Laurentius Salvius: Holmiae, ii, 824 pp. 1758. Available from: https://doi.org/10.5962/bhl.title.542

58. Longhurst A. Ecological Geography of the Sea, seconded. Academic Press. 2007.

59. Majiedt PA, Holness S, Sink KJ, Reed J, Franken M, van der Bank MG, Harris LR, Adams L, Perschke M, Miza SA, Currie J, Dunga LV. Chapter 4: Pressures on Marine Biodiversity. In: Sink KJ, van der Bank MG, Majiedt PA, Harris LR, Atkinson LJ, Kirkman SP, Karenyi N (eds). 2019. South African National Biodiversity Assessment 2018 Technical Report Volume 4: Marine Realm. South African National Biodiversity Institute, Pretoria. South Africa. 2019. Available from: http://hdl.handle.net/20.500.12143/6372

60. Marenzeller von E. Steinkorallen. Wissenschaftliche Ergebnisse der Deutschen Tiefsee-Expedition auf dem Dampfer Valdivia. 1904; 1898–1899 7(3): 261–318.

61. McQuaid KA, Bridges AEH, Howell KL, Gandra TBR, de Souza V, Currie JC, Hogg OT, Pearman TRR, Bell JB, Atkinson LJ, Baum D, Bonetti J, Carranza A, Defeo O, Furey T, Gasalla MA., Golding N, Hampton SL, Horta S, Jones DOB, Lombard AT, Manca E, Marin Y, Martin S, Mortensen P, Passadore C, Piechaud N, Sink KJ, Yool A. Broad-scale benthic habitat

classification of the South Atlantic. Progress in oceanography. 2023; 103016. https://doi.org/10.1016/j.pocean.2023.103016

62. Milne-Edwards, H, Haime J. Recherches sur les Polypiers, deuxième mémoire: Monographie des Turbinolides. Ann Sci Nat Zool. 1848a; 3(9): 211–344.

63. Naumann MS, Orejas C, Ferrier-Pagès C. Species-specific physiological response by the cold-water corals *Lophelia pertusa* and *Madrepora oculata* to variations within their natural temperature range. Deep Sea Res Part II: Top Stud Oceanogr. 2014; 99, pp.36-41.

64. Oakham V. Deep-Sea Coral Reefs: Distribution, Ecology and Anthropogenic Impacts. 2009.

65. O'Hara TD, Rowden AA, Bax NJ. A southern hemisphere bathyal fauna is distributed in latitudinal bands. Current Biology. 2011; 21(3):226-30.

66. Olbers JM. Taxonomy, biodiversity and biogeography of the brittle stars (Echinodermata: Ophiuroidea) of South Africa. PhD. Thesis. University of Cape Town. 2016. Available from: http://hdl.handle.net/11427/22906

67. Orejas C, Wienberg C, Titschack J, Tamborrino L, Freiwald A, Hebbeln D. *Madrepora oculata* forms large frameworks in hypoxic waters off Angola (SE Atlantic). Scientific Reports. 2021;11(1):15170.

68. Owens J. *Letepsammia franki*, a new species of deep-sea coral (Coelenterata: Scleractinia: Micrabaciidae). Proc Biol Soc. 1994; 107(4): 586–590.

69. Reygondeau G. Current and future biogeography of exploited marine exploited groups under climate change. In Predicting Future Oceans: Sustainability of Ocean and Human Systems Amidst Global Environmental Change. Elsevier Inc. 2019. https://doi.org/10.1016/B978-0-12-817945-1.00009-5

70. Reygondeau G, Dunn D. Pelagic biogeography. Encyclopedia of Ocean Sciences. 2018 ; 588–598. https://doi.org/10.1016/B978-0-12-409548-9.11633-1

71. Roberts J, Wheeler A, Freiwald A. Cairns S. Cold-water corals: the biology and geology of deep-sea coral habitats. Cambridge University Press. 2009.

72. Richter DJ, Watteaux R, Vannier T, Leconte J, Frémont P, Reygondeau G, Maillet N, Henry N, Benoit G, Da Silva O, Delmont TO. Genomic evidence for global ocean plankton biogeography shaped by large-scale current systems. Elife. 2022;11:e78129.

73. Sayre R, Wright D, Breyer S, Butler K, Van Graafeiland K, Costello M, Harris PT, Goodin K, Guinotte J, Basher Z, Kavanaugh M, Halpin P, Monaco M, Cressie N, Aniello P, Frye C, Stephens D. A three-dimensional mapping of the ocean based on environmental data. Oceanography. 2017; 30(1), 90–103. https://doi.org/10.5670/oceanog.2017.116

74. Schouten M, de Ruijter W, Van Leeuwen P, Lutjeharms J. Translation, decay and splitting of Agulhas rings in the southeastern Atlantic Ocean. J Geophys Res. 2000; 105(C9), 21913-21925p. https://doi.org/10.1029/1999JC000046

75. Shannon LV. The Benguela ecosystem. I: Evolution of the Benguela physical features and processes. Oceanogr Mar Biol. 1985; 23, pp.105-182.

76. Sheppard CRC, Sheppard ALS. Corals and coral communities of Saudi Arabia. Fauna of Saudi Arabia. 12: 1-170. 1991.

77. Sink K, van der Bank M, Majiedt P, Harris L, Atkinson L, Kirkman S, Karenyi N (eds). In: South African National Biodiversity Assessment 2018 Technical Report Volume 4: Marine Realm. South African National Biodiversity Institute, Pretoria. South Africa. 2019. Available from: http://hdl.handle.net/20.500.12143/6372

78. Sink KJ, Adams LA, Franken M-L, Harris LR, Currie J, Karenyi N, Dayaram A, Porter S, Kirkman S, Pfaff M, van Niekerk L, Atkinson LJ, Bernard A, Bessinger M, Cawthra H, de

Wet W, Dunga L, Filander Z, Green A, Herbert D, Holness S, Lamberth S, Livingstone T, Lück-Vogel M, Mackay F, Makwela M, Palmer R, Van Zyl W, Skowno A. Iterative mapping of marine ecosystems for spatial status assessment, prioritization, and decision support. *Front. Ecol. Evol*. 2023; 11:1108118. doi: 10.3389/fevo.2023.1108118

79. Spalding M, Fox H, Allen G, Davidson N, Ferdaña Z, Finlayson M, Halpern BS, Jorge M, Lombana A, Lourie S, Martin,K, Mcmanus E, Molnar J, Recchia C, Robertson J. Marine Ecoregions of the World: A Bioregionalization of Coastal and Shelf Areas. BioScience. 2007; 57(7), 573–583. https://doi.org/10.1641/B570707.

80. Spengler L. Beskrivelse over et ganske besynderligt Corall-product, hvilket man, indtil dets Sloegt noermere bestemmes, kunde kalde en Snekke-Madrepore (Madrepora cochlea). Nye Saml. Danske Videnskebernes Selskab Skrifter 1: 240–248. 1781.

81. Summers N, Watling L. Upper Bathyal Pacific Ocean biogeographic provinces from octocoral distributions. Progr Oceanogr. . 2021; 191, 102509. https://doi.org/10.1016/j.pocean.2020.102509\

82. Thandar S. Zoogeography of the southern African echinoderm fauna. S Afric J Zool. 1989; 24(4), 311–318. https://doi.org/10.1111/j.1365-2699.2004.01144.x

83. United Nations. Revised Roadmap for the UN Decade of Ocean Science for Sustainable Development. 2018. Available online at: http://www.fao.org/3/CA0463EN/ca0463en.pdf

84. United Nations. Revised draft text of an agreement under the United Nations Convention on the Law of the Sea on the conservation and sustainable use of marine biological diversity of areas beyond national jurisdiction, Intergovernmental conference on an international legally binding instrument under the United Nations Convention on the Law of the Sea on the conservation and sustainable use of marine biological diversity of areas beyond national jurisdiction (fourth session, New York, 23 March–3 April 2020). UNGA: New York. 2019.

85. United Nations Environment Program Convention on Biological Diversity. Zero draft of the post-2020 global biodiversity framework. 2020. Available online at: https://www.cbd.int/doc/c/efb0/1f84/a892b98d2982a829962b6371/wg2020-02-03-en.pdf

86. Vaughan T. A critical review of the literature on the simple genera of the Madreporaria Fungida, with a tentative classification. Proc U S Natl Mus. 1905; 28(1401): 371–424. https://doi.org/10.5479/si.00963801.1401.371

87. Verrill A. Classification of polyps (extract condensed from Synopsis of the Polyps and Corals of the North Pacific Exploring Expedition under Commodore C. Ringgold and Captain John Rodgers, U.S.N.). Communications of the Essex Institute 4: 145–152. 1865. Avaialble from: https://www.biodiversitylibrary.org/page/34752272.

88. Waters J. Driven by the West Wind Drift? A synthesis of southern temperate marine biogeography, with new directions for dispersalism. J Biogeogr. 2008; 35(3), 417–427. https://doi.org/10.1111/j.1365-2699.2007.01724.x

89. Watling L, Guinotte J, Clark MR, Smith CR. A proposed biogeography of the deep ocean floor. Progress in Oceanography. 2013;111:91-112.

90. Watling L, Lapointe A. Global biogeography of the lower bathyal (700–3000 m) as determined from the distributions of cnidarian anthozoans. Deep Sea Res Part I: Oceanogr Res Pap. 2022; p.103703. https://doi.org/10.1016/j.dsr.2022.103703

91. Woolley S, Foster S, Bax N, Currie J, Dunn D, Hansen C, Hill N, O'Hara T, Ovaskainen O, Sayre R, Vanhatalo J, Dunstan P. Bioregions in Marine Environments: Combining Biological and Environmental Data for Management and Scientific Understanding. BioScience. 2020; 70(1), 48–59. https://doi.org/10.1093/biosci/biz133

92. WoRMS Editorial Board. World Register of Marine Species. Available from https://www.marinespecies.org at VLIZ. 2023. Accessed 2023-02-17. doi:10.14284/170

93. Zezina ON. Biogeography of the bathyal zone. In Advances in Marine Biology 1997 (Vol. 32, pp. 389-426). Academic Press.

## Supporting information

**Appendix A:** Excel

**Appendix B:** Draftsman's plot.

**Figure 2**. Draftsman's plot showing the interrelations between depth and the longitudinal groups identified at by the k-R cluster mean analysis.

**Appendix C:** Excel

**Appendix D**: Histograms

**Figure 5**. Simulated distribution/histogram of the test statistic R under the null hypothesis that there are "no differences" in coral patterns within each condition: **TOP**. krLong groups with observed data at R=0.05 and p= 0.001; **BOTTOM**. z-level (depth) with observed data at R = 0.072 and p = 0.001.

**Figure 6**: Simulated distribution/histogram of the test statistic R under the null hypothesis that there are "no differences" in coral patterns within depth zones (R = 0.105, p = 0.001).

Figure

Click here to access/download;Figure;Figure 1.tif

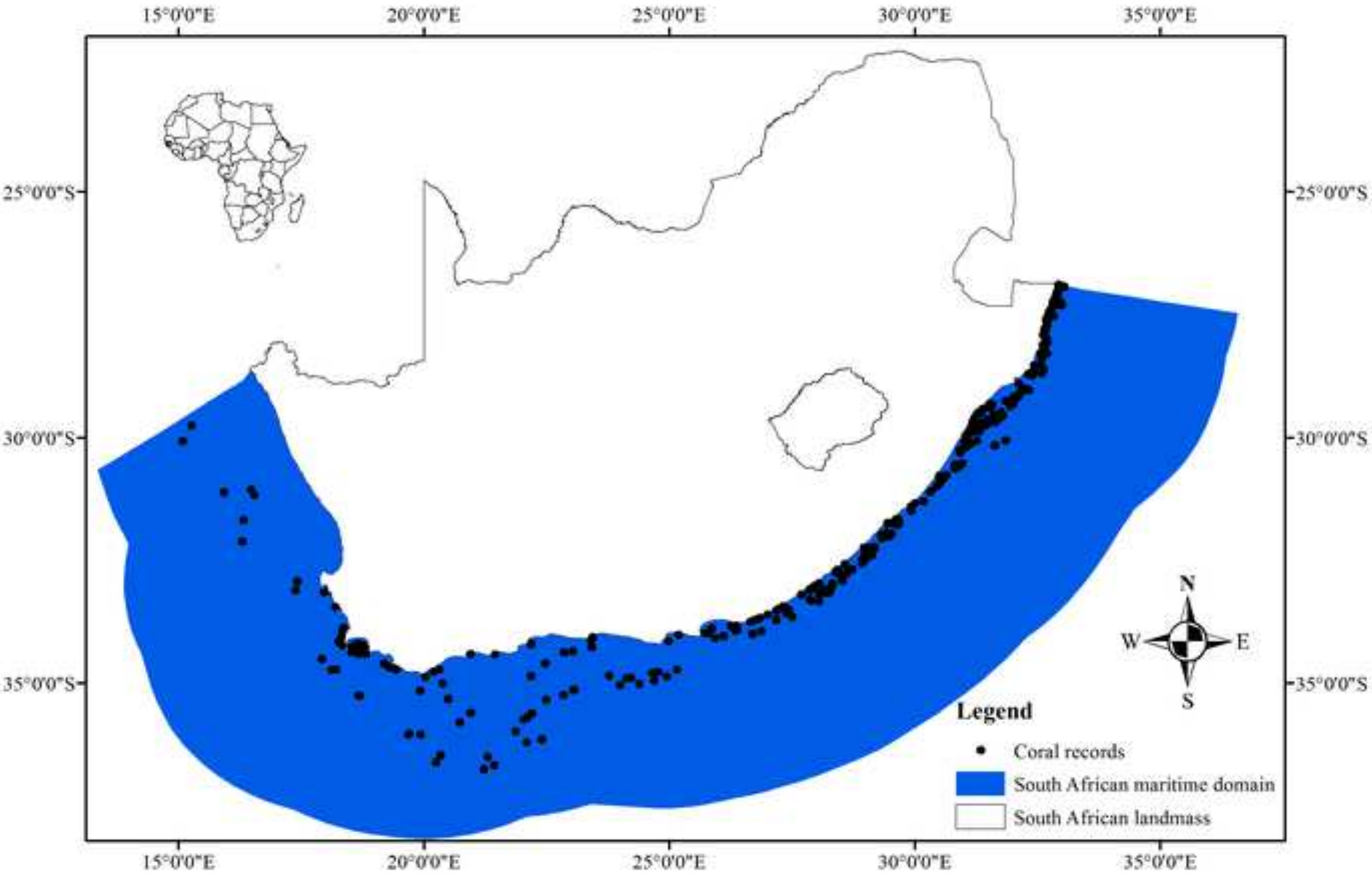

Figure Click here to access/download;Figure;Figure 3.tif

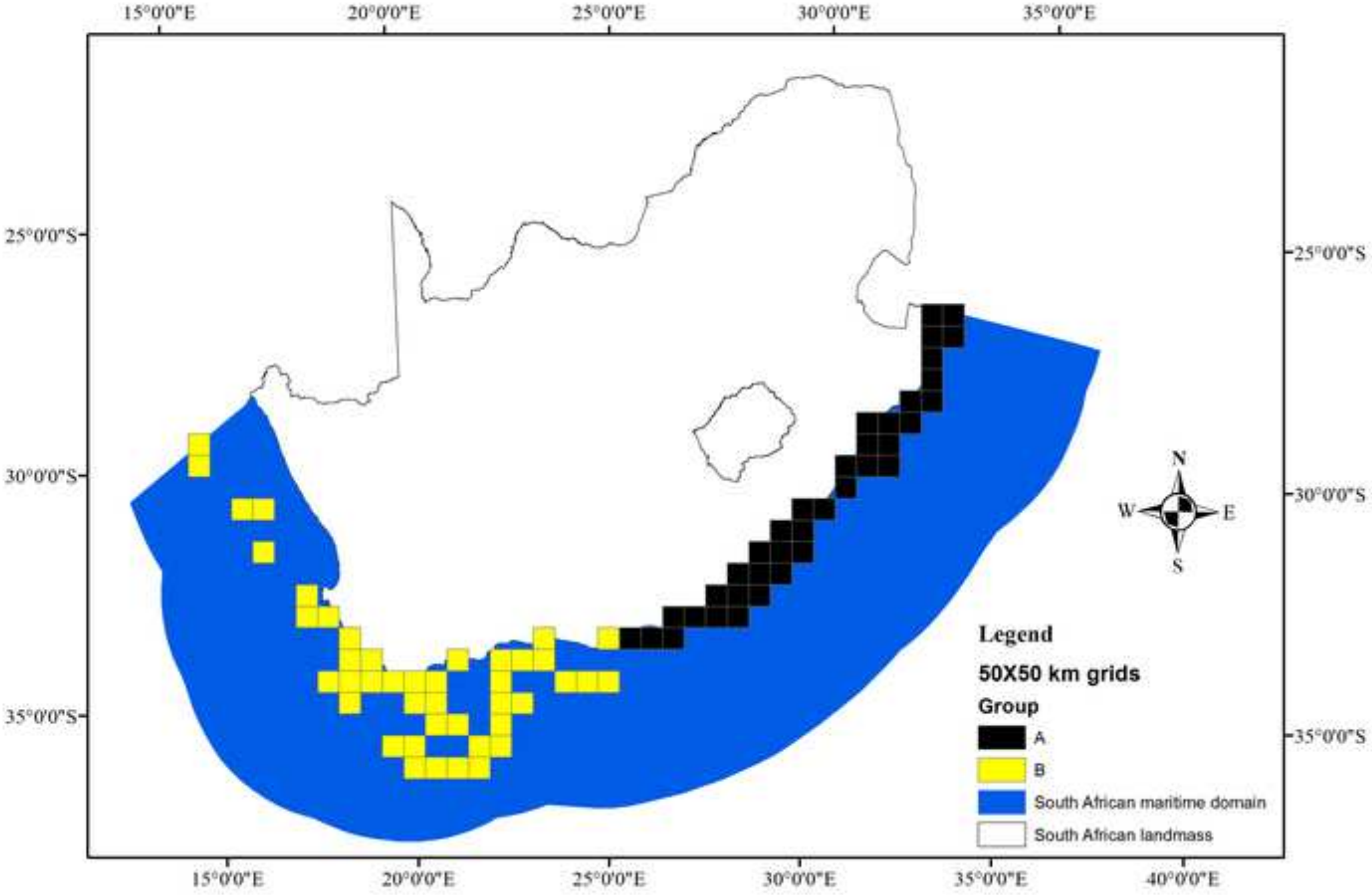

Figure

Click here to access/download;Figure;Figure 4.tiff

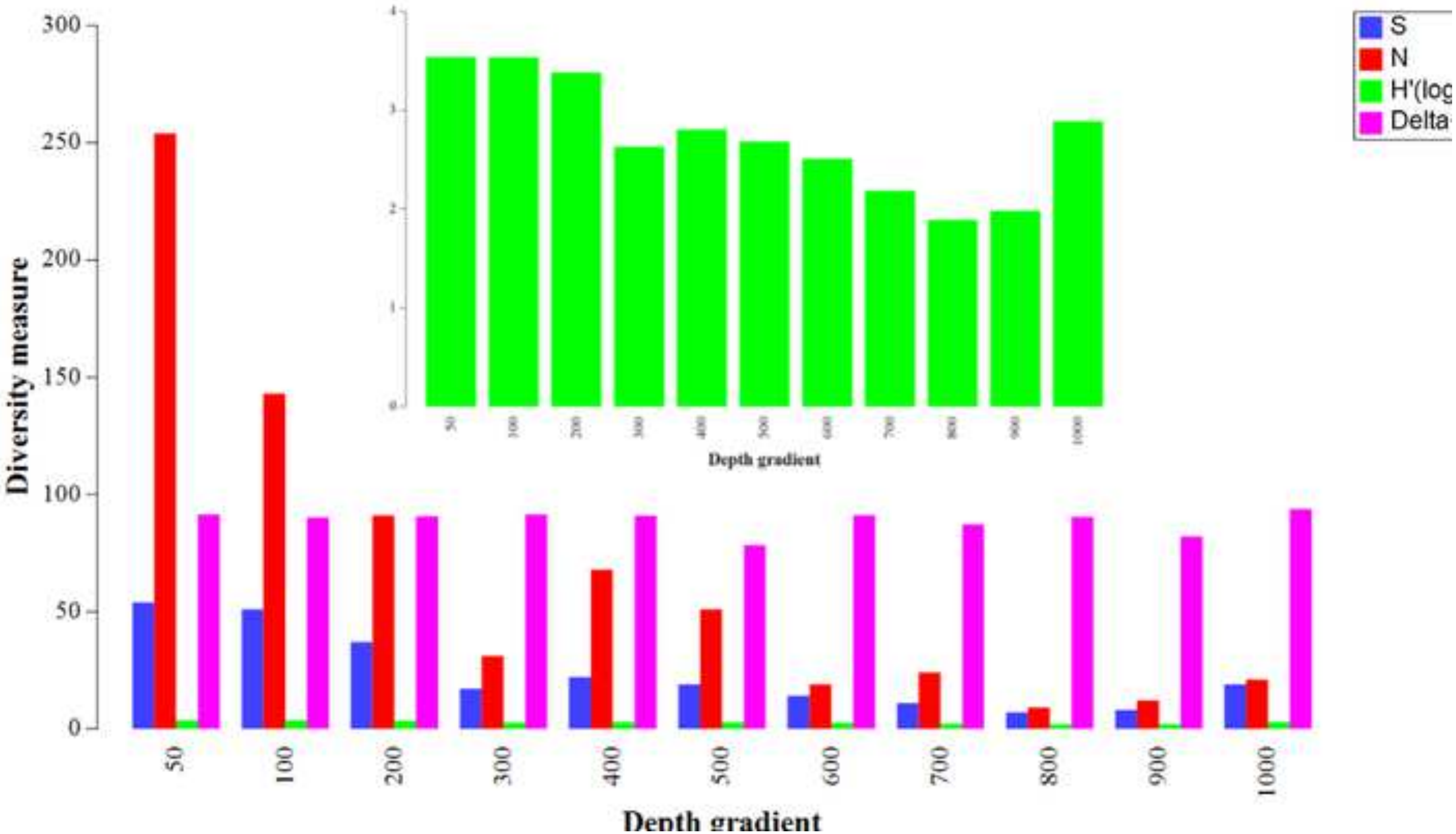

Click here to access/download;Figure;Figure 8.tif

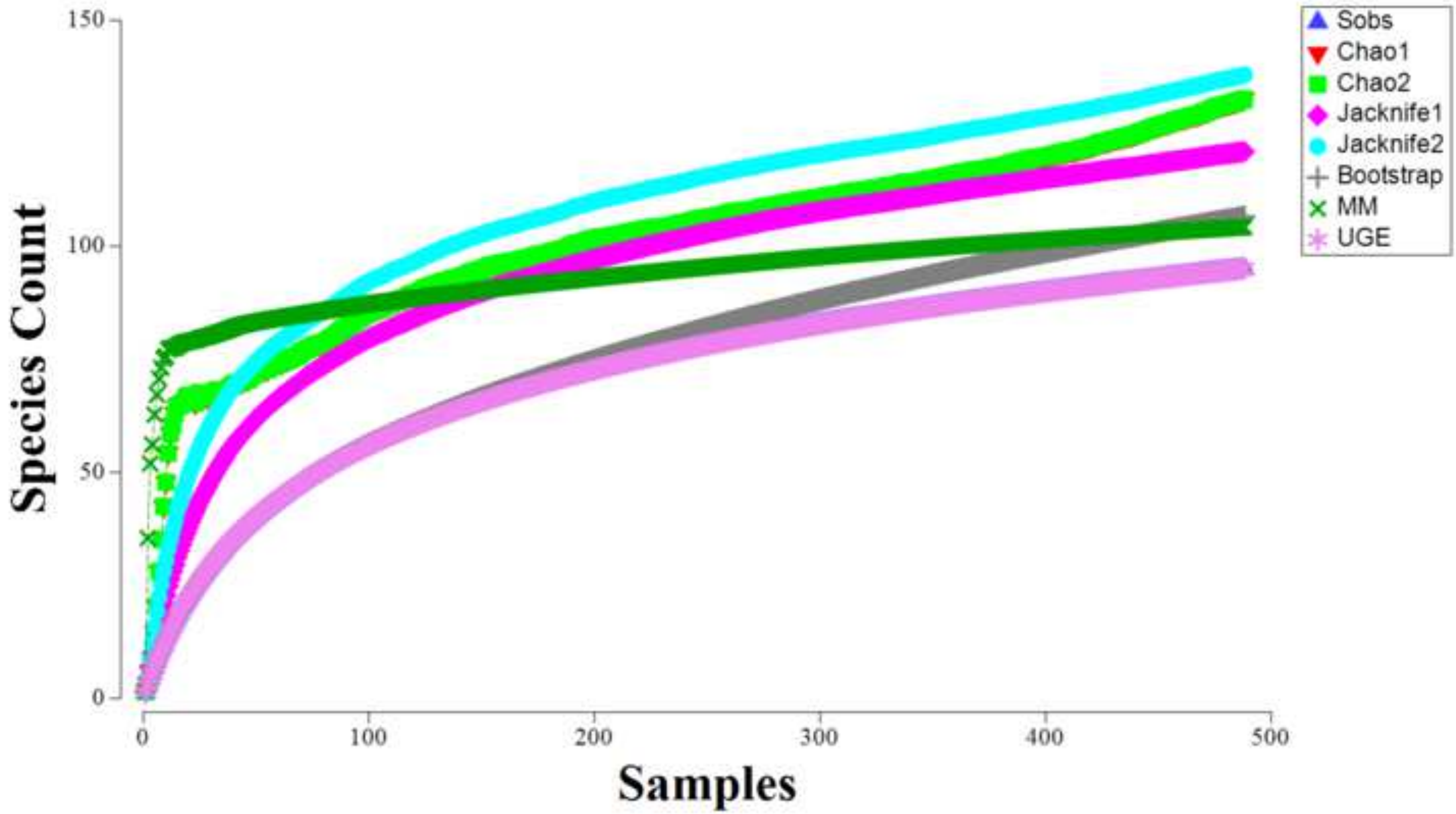

Figure Click here to access/download;Figure;Figure 7.tif

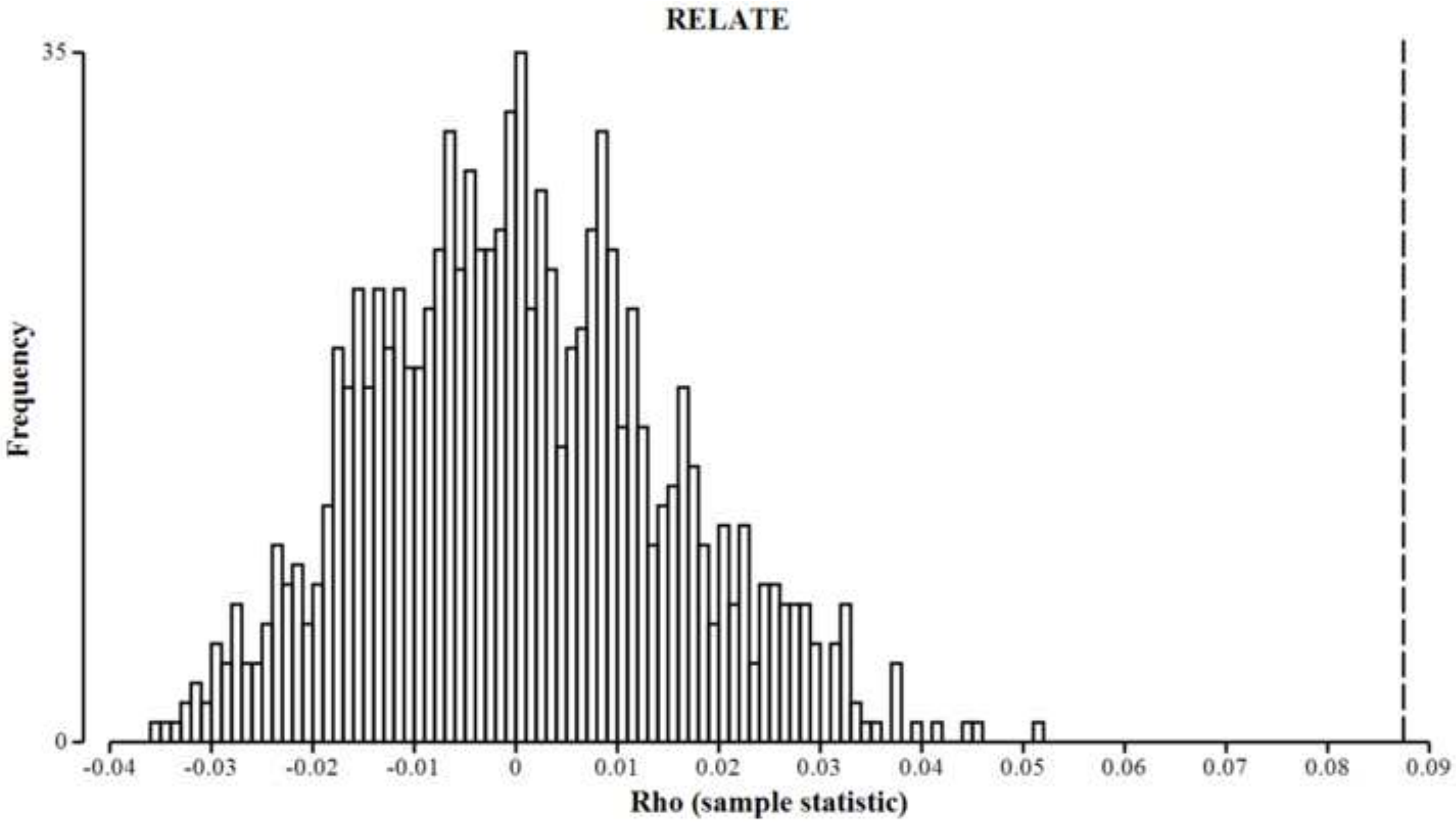

Supporting figure

Click here to access/download
**Supporting Information**
Appendix D_Figure 5. TOP.tif

Supporting figure

Click here to access/download
**Supporting Information**
Appendix D_Figure 5. Bottom.tif

Supporting figure

Click here to access/download
**Supporting Information**
Appendix B_Figure 2.tif

Supporting figure

Click here to access/download
**Supporting Information**
Appendix D_Figure 6.tif

excel

Click here to access/download
**Supporting Information**
Appendix A_biopaper_plos paper.xlsx

excel

Click here to access/download
**Supporting Information**
Appendix C_biopaper_plos paper.xlsx
