# Supplementary material for: Diversity patterns of the South African azooxanthellate scleractinians (Cnidaria: Anthozoa), with considerations of environmental correlates
Source: PLoS One. 2024 Aug 8;19(8):e0296188. doi: 10.1371/journal.pone.0296188 (PMC11309500; doi:10.1371/journal.pone.0296188)
Supplement: Attachment — Submitted filename: Rebuttal.docx [file pone.0296188.s004.docx]

Nelson Mandela University

University Way

Summerstrand

Gqeberha

6019

South Africa

16 November 2023

PLOS ONE

1265 Battery Street, Suite 200

San Francisco,

CA 94111

United states of America

Dear Dr Carlo Nike Bianchi (Academic Editor)

**Re: Rebuttal Letter for Manuscript ID [PONE-D-23-17495]**

**Initial title of manuscript**: The application of historic sample-specific variables in evaluating the biodiversity patterns of the South African azooxanthellate scleractinians (Cnidaria: Anthozoa).

**Revised title of manuscript**: Diversity patterns of the South African azooxanthellate scleractinians (Cnidaria: Anthozoa), with considerations of environmental correlates.

I trust this correspondence finds you in good health. I want to convey my appreciation for the meticulous evaluation and feedback given by the reviewer(s) regarding the submitted manuscript to PLOS ONE. The dedication of both the reviewer and the editorial team in assessing this work and providing constructive suggestions is genuinely valued.

We have carefully examined the reviewers' comments and suggestions, and I wish to illustrate our responsiveness to their concerns. Below, we have delineated each major concern along with its corresponding response.

Regards,

Zoleka Filander (on behalf of co-authors)

**Reviewer 2**

**Reviewer #2 overview**:  a large number of revisions and corrections have been made to this version which now reads much more clearly (although still long in relation to the data content in my opinion!

**Authors response**: We thank the reviewer for the suggestion to compress the contents within the manuscript. This has prompted improved referencing of the methodology and the elimination of redundant/repetitive information. The manuscript has undergone rearrangement, resulting in a reduction of the text from 33 to 31 pages. To enhance representation and reference convenience, Figure 2 has been integrated with the draftsman's plot results, and the permutation histogram of the RELATE results has been omitted. These comprehensive edits have necessitated a title change, aligning more accurately with the manuscript's refined content.

**Reviewer #2 comment**: I spotted only a small number of points that still need correction as follows ( indicated by line number):

- 83 - i presume you mean 'since the 1700's'
- 98- it is not necessary of useful to insert 'i.e. ' when detailing a set of items in brackets. This happens many times in MS and should be removed
- 101-103 i suggest merging these two sentences instead or repeating the reference
- 149 - data are pleural ( of datum), so 'are' not 'is'
- 288- space needed before m
- Refs 8-9 words in the one title given with caps, in the other in lower case - which is correct format?
- ref 27 - in different font to others
- 587 - there is a spare M in this line
- 596 full stop needed after title
- 623 Seconded? do you mean Second Edition?
- 643 i think title is Research not Res, which is abbreviation
- 644 no journal given for this ref
- Ref 92 why are words capitalised in this title
- Figs 1 and 2 could easily be combined by adding blacks to the first figure

**Authors response**: All the aforementioned suggestions have been incorporated, with revisions made to rectify these errors, and the manuscript has undergone significant editing..
